# Supplementary material for: Are carbon emissions trading and green financial instruments synergistic? -Comprehensive quantitative research based on content analysis
Source: PLoS One. 2024 Mar 7;19(3):e0298601. doi: 10.1371/journal.pone.0298601 (PMC10919601; doi:10.1371/journal.pone.0298601)
Supplement: S1 Data — (ZIP) [file pone.0298601.s001.zip › Data/地级市Z_F工作报告.zip!/╝¬┴╓/░╫╔╜╩╨/└┤╫╘╣1⁄2╓┌║┼í╛┬φ┐╦╩2╛▌═°í┐_2015.docx]

各位代表：

　　现在，我代表市政府向大会报告工作，请予审议，并请列席人员提出意见。

　　一、2014年工作回顾

　　过去一年，面对复杂多变的发展形势和经济下行的巨大压力，我们在省委、省政府和市委的正确领导下，在市人大、市政协的监督支持下，以转型发展为主线，以富民强市为目标，凝心聚力，攻坚克难，坚决贯彻实施稳增长、促改革、调结构、惠民生一系列重大举措，全力推进白山经济社会持续健康稳定发展。

　　（一）调结构、促转型，经济发展稳中有进。预计全市实现地区生产总值720亿元，增长7%；固定资产投资600亿元，增长16%；地方级财政收入44.3亿元，其中税收增长3%。项目带动作用增强。3千万元以上项目开复工500个，完成投资460亿元。其中亿元以上241个，完成投资395亿元；“333”产业项目300个，完成投资280亿元。新开工恒大年产500万吨矿泉饮品、长白山国际度假区水上乐园等249个项目，竣工投产喜丰节水器材、蓝锐LED等130个项目，谋划297个重点项目，其中接替项目158个，滚动谋划亿元以上项目110个。新型工业化扎实推进。预计实现规上工业增加值385亿元，增长6.5%。工业技改投资增长15%，用电量增长9%。积极推进产学研合作平台建设，设立全市首家院士工作站，新增合兴、峰泰源两件中国驰名商标。投产新产品56个，新产品产值率达到9%。获批省级“双十”项目2项、科学技术奖3项，创新型科技企业达到11户。万元工业增加值综合能耗下降8.5%，超额完成省定目标。整装勘查累计投资6亿元，新增金、银、铜、铁、硅藻土储量50吨、200吨、50万吨、2.8亿吨和1.2亿吨，成功吸引了东北亚、盼盼等投资主体实施深加工、新产品项目75个。深化与恒大、农夫山泉等战略合作，恒大冰泉“一处水源供全球”战略开创长白山矿泉水出口世界新时代，全市矿泉饮品产能翻番达到800万吨。医药产业实力增强，施慧达药业被评为国家知识产权优势企业，修正长白山药业竣工投产。全市9处煤矿复产复工，77户“双停”企业启动生产。特色农业效益凸显。预计实现农业增加值61亿元，增长4.5%。大力发展棚膜经济，新增标准化棚膜3千亩，全市蔬菜自给率达到61.2%。特色产业快速增长，预计产值达到103亿元，增长21%。人参食品达到63个，人参皂苷、多肽等核心竞争力产品达到7个，林下参、西洋参获批国家地理标志保护产品。与天津渤海商品交易所签订 “长白山农特珍品交易中心”战略合作框架协议，我市农特产品开始融入全球24小时电子交易平台。大祺农超对接成功打入北京物美、长春欧亚等大型连锁超市。现代服务业提档升级。预计实现服务业增加值230亿元，增长7.5%。旅游服务水平和接待能力不断提高，接待国内外游客650万人次，旅游社会总收入79.8亿元，增长15%和25%。积极发展电子商务，首批入驻淘宝网企业80户、名优特产品400个品种。中药材流通追溯体系国家试点项目建成运行，改造升级18家农贸市场。实现社会消费品零售总额244亿元，增长12%。

　　（二）促改革、扩开放，发展活力明显提升。各项改革深入推进。白山国家主体功能区示范试点获批。改变以GDP为核心的考核评价机制，编制绿色转型评价指标体系。深化政府机构改革，新组建市卫计委和食药监局，撤并市畜牧局和外事办，全面接收工商、质监职能，各县（市、区）政府机构改革方案通过审批。加大行政审批制度改革力度，启动权力清单制度工作。积极深化集体林权制度改革，集体林地所有权发证222万亩，流转22万亩。推进医药卫生体制改革，基层医疗卫生机构全部实现基本药物零差率销售和药品网上集中采购，抚松、临江被列为全国县级公立医院综合改革试点县。深化户籍制度改革，合理有序引导农业人口向城镇转移。金融改革有新进展，组建靖宇农商行和抚松榆银、靖宇乾丰村镇银行。开放活力明显增强。积极“走出去”、“请进来”，有针对性组织叩门招商。恒大、香雪人参等一批产业项目进入实质洽谈，正大千万吨矿泉水、台湾允丰收科技生物肥等项目正式签约。实际引进市外资金500亿元，增长15%；实际利用外资2.27亿美元，增长10%。新增外贸进出口经营权企业25户，长白山人参国家级外贸转型升级示范基地正式启动，长白国家重点开发开放试验区纳入新一轮振兴东北规划。积极拓展进出口市场，实现外贸总额2.9亿美元。民营经济加快发展。全市民营经济实现主营业务收入1400亿元，增长19.3%，从业人员达到35万人，增长5.1%，民企和个体工商户分别达到7410户和6.8万户。为民企减免税费1.3亿元，金融机构为民企贷款余额165.5亿元，增长14.8%。市创业大厦和中小企业公共服务平台投入使用，启动助保金池贷款业务。我市被评为全省突出发展民营经济先进单位。

　　（三）抓建设、树形象，城乡环境持续改观。基础设施日益完善。实施畅通、净化、供水保障、气化白山和物业整治五大工程，新改建浑江大街等23条街路，光明街、青松路排水工程以及南岭高位水池和南山净水厂竣工，二次供水管网改造顺利推进。市区新增燃气用户6千户，燃气普及率提高到35.2%。营松、鹤大高速完成年度建设任务，辉白高速完成工程招投标，征地顺利推进，白临高速完成工可研，长松岭隧道贯通，白山至三道沟公路完成部分主体工程，新调整升级国道3条737.2公里，省道6条351.7公里，干线路网进一步完善。实施农村饮水安全、浑江干流整治、中小河流治理和病险水库加固四大水利工程，矿泉湖水利枢纽完成投资5千万元，电站厂房主体封闭。人居环境明显提升。启动创建国家级园林城，申报中国人居范例奖。市中心区栽植乔灌木、花卉180余万株，完成一江两岸亮化及41条街巷照明工程。深入开展市容市貌专项整治，实施全天候保洁，城乡环境进一步改善。靖宇、江源等中心城镇建设有序推进。完成58个省级示范村项目和120个村集中整治。生态文明建设成效显著。实施重点流域污染防治工程，完成7个松花江流域治理项目，各县（市、区）污水处理厂全部投入运行，主要污染物削减任务全部完成，提前一年实现“十二五”大气污染物减排目标。推进水生态系统保护与修复，治理水土流失面积16万亩。深入实施天保工程，清收林地3.2万公顷，还林2.3万公顷，3年任务当年完成过半。我市被评为2014年度中国生态文明价值城市，抚松、临江入选“中国深呼吸小城100佳”，红土崖河湿地公园通过国家级评审，五道沟国家级湿地公园获批，白山原麝保护区晋升为国家级自然保护区。

　　（四）惠民生、促和谐，群众生活不断改善。持续加大民生投入，年初承诺的37件民生实事有31件完成，其中超额完成8件，取消1件，其余5件正在有效推进。积极开展就业援助活动，建成7个就业困难人员大市场和23个农民工创业示范基地，城镇新增就业4.8万人，农村劳动力转移就业12.2万人。五大险种进一步提标扩面，城乡低保实现应保尽保。棚户区开工建设30.5万平方米，当年回迁安置2442户，改造D级危房12.5万平方米。城乡居民人均可支配收入分别达到26690元和10150元。推进教学改革，规范办学行为，落实助贫政策，促进教育公平。公共卫生和医疗服务保障体系进一步完善，重点传染病发病率明显低于全省平均水平。食品药品安全监管不断加强。落实生育调整政策，加强计生管理和服务。公益文化、群众体育活动广泛开展。完善领导干部接访下访制度，有效化解原红旗电影院区域改造等187件信访积案。构建立体化治安防控体系，农村视频监控系统覆盖率达到70%。公安信息化建设走在全省前列，刑事犯罪率大幅下降，命案现案破案率继续保持全省第一。深入开展平安白山建设，基层平安建设成效明显。扎实推进兴边富民工程和五边战略，长白县连续第六次被评为全国民族团结进步模范县。推进“四化融合”、“三位一体”安全监管防控体系建设，大力开展春季行动、夏季攻坚、重大火灾隐患集中整治和秋冬会战等专项行动，安全生产形势趋好。粮食应急储备安全体系进一步完善，市粮食局被评为全国粮食系统先进集体。连续35年无重大森林火灾。

　　（五）转作风、提效能，发展环境全面优化。深入开展党的群众路线教育实践活动，查找 “四风”突出问题，扎实开展“三公”经费、公车治理等32项专项整治，完成119项整改落实和制度建设任务。推进简政放权，审批项目清理38项，下放9项，市本级精简到216项，其中即办件110件，承诺件106件，编制审批目录并向社会公开。深入实施“权力入笼”工程，开展4场大型政行风“电视听政”，廉政建设和政行风建设明显加强。集中开展软环境整治，加强重点领域专项执法监察，查处涉软和“四风”案件140件。自觉接受人大法律监督、工作监督和政协民主监督，办理人大代表建议58件、政协委员提案66件，面复率和满意率100%。

此外，统计、水文、气象、人防、供销、地震、外事、地方志、档案、老龄、残联、妇女儿童、民族宗教、住房公积金、民兵预备役等工作都为全市经济社会发展作出了积极贡献。

各位代表，过去的一年是我们艰难爬坡、奋进前行的一年。面对困难挑战，能够取得这样的成绩来之不易。这是市委总揽全局、坚强领导的结果，是市人大、市政协有效监督、全力支持的结果，是全市各族人民戮力同心、奋勇拼搏的结果。在此，我代表市政府，向全市各族人民，向驻白山人民解放军指战员、武警部队官兵、公安司法干警、中省属企事业单位，向各民主党派、工商联、人民团体、无党派人士、离退休老同志，向关心和支持白山发展的各界朋友，致以崇高的敬意和衷心的感谢！

在总结成绩的同时，我们也清醒地认识到，发展中还面临不少困难和问题：转型发展迫在眉睫，经济结构不优，资源产业化步伐不快，接替产业不强；改革任务艰巨，一些体制机制性障碍亟待破解，软环境还有待进一步改善；区位条件不优，开放层次不高；基础设施和社会事业欠账较多，公共服务能力不强，一些群众的利益诉求还没有得到很好地解决。这些问题事关全局，我们一定高度重视，善做善成、倾力解决。

　　二、2015年工作安排

　　今年是“十二五”收官之年和“十三五”谋划之年，也是全面深化改革、推进依法治市、加快转型发展承前启后的重要一年，多元挑战并存、多重任务叠加、多种机遇汇集。当前，我国经济正处于增速换挡回落、结构发生全面深刻变化、从要素驱动转向创新驱动的新阶段，进入以中高速、优结构、新动力、多挑战为主要特征的新常态，经济韧性好、潜力足、回旋余地大，为持续健康发展提供了有利条件。随着全面深化改革不断释放政策红利，工业化、城镇化持续推进，特别是国家出台新一轮振兴东北政策，深入推进能源生产和消费革命，东北东部经济带即将上升为国家战略，省委、省政府审时度势提出建设吉林东部绿色转型发展区的新战略，必将为白山转型跨越、绿色崛起注入强大动力。面对新的发展形势，我们必须牢固树立必胜信心，科学规划“十三五”工作，全力以赴在抢抓机遇中争创优势，在开拓创新中奋发有为，在深化改革中激发活力，在绿色转型中抢占先机，努力走出一条生态与经济良性互动、人与自然和谐相处的新路子。

　　今年政府工作总体要求是：全面贯彻党的十八届三中、四中全会精神以及省委、省政府和市委决策部署，坚持稳中求进总基调，突出绿色转型主旋律，主动适应经济新常态，切实提高经济增长质量和效益，狠抓改革攻坚、扩大对外开放、统筹推进四化、建设生态文明、强化民生保障、推进依法行政，全力建设吉林东部绿色转型发展先行区。

　　综合考虑各种因素，全市经济社会发展主要预期目标是：地区生产总值增长7%，固定资产投资增长15%，地方级财政收入增长5%，社会消费品零售总额增长10%，城乡居民人均可支配收入分别增长6%和8.5%，城镇登记失业率控制在4.5%以内，单位GDP综合能耗降低3%，二氧化碳排放降低3.73%。

　　围绕上述目标任务，要突出抓好七方面重点工作：

　　（一）着力在产业集群上下功夫，强壮绿色转型支柱。立足资源禀赋和优势特色，大力实施存量经济绿色化改造和增量经济绿色化构建，加快形成集约集群发展的绿色产业体系。

　　一是矿产新材料产业。把矿产新材料产业作为调结构、促转型的“第一接力棒”，全年产值力争突破550亿元，增长20%，努力打造我市率先突破千亿级的产业集群。深入实施整装勘查，投资1.5亿元推进20个重点勘探项目，推动与三棵树等企业勘查开发合作，鼓励各种所有制形式企业通过并购等方式参与。加速找矿成果转化，对具备开采能力、技术条件成熟、能够形成产业的矿产资源尽快开发投产。研发高科技含量、高附加值产品，打造硅、镁、铁、硅藻土四大产业链条，加快建设新材料产业园区，创建国家级战略性新兴产业示范区。二是矿泉饮品产业。整合资源，坚持招大商、引名企、建名厂、育名牌，开发中高端产品市场，集中力量扩大释放产能，打响长白山天然矿泉水地域品牌，产值力争翻一番，突破120亿元。加快推进恒大矿泉水基地、农夫山泉四期、生命人寿保险100万吨麦饭石矿物质水等项目，带动矿泉水产业实现更大突破。积极发展围水包装产业，形成以包装用纸、纸箱和彩印等产品为主，与矿泉饮品产业相互链接、协调配套、相互促进的新型包装产业链。三是人参产业。抢抓被列入国家重点培育发展接替产业的有利机遇，抓好三大园区、四大基地、五大中心建设，全年实现产值210亿元，增长10%。加快推进广东太安堂、广药东阿、吉林加一等项目，创建人参高新技术园。推广28项优质安全栽培技术，林下参、非林地栽参分别发展到55万亩、600万平方米，GAP面积稳定在2千万平方米。健全质量安全可追溯体系，完善电子交易平台，推动万良人参市场省部共建国家级重点专业市场，打造集生产、贸易、文化为一体的世界人参集散地。四是旅游产业。立足世界顶级生态资源优势，充分发挥长白山国际度假区龙头作用，推进鲁能漫江生态旅游开发、广泽旅游度假区、长白山关东文化园等一批项目，开发“五彩旅游”精品景区和线路，突出区域内旅游节点规划建设，完善配套基础设施，推进旅游产品开发，提升行业服务水平，着力构建“一核三带六城”为支撑的环长白山生态旅游圈，打造水陆空一体的立体化旅游新格局。全年接待国内外游客750万人次，旅游社会总收入突破百亿元，分别增长15%和25%。五是医药产业。深入落实省政府关于加快推进医药健康支柱产业实施意见，强化科技支撑，加大研发力度，培育龙头企业，做强重点领域，全年实现产值200亿元，增长20%。积极推进生人参胶囊、中药注射液及饮片等项目，加快人参一类抗癌等药物市场化进程，扶持以修正、葵花为代表的中药现代化企业，以施慧达为代表的化药和生物药企业，加快形成以中药饮片、中药提取物、中成药、中药保健、化学药及生物制药五大产品链为主体的产业体系。六是特色农产品产业。坚持走非均衡、差异化发展之路，大力发展生态效益型精品特色农业，全年实现产值110亿元，增长6.8%。建设浑江食用菌、江源蓝莓、靖宇中药材等6个农业产业化园区，新培育2户国家级休闲农业旅游企业，助农致富6万户以上。加快发展长白山森林黑猪、优质肉牛、蜜蜂、梅花鹿和特种经济动物等特色牧业经济，推进大山合等10个3千万元以上产业化项目，形成林果、食用菌、山野菜、特色牧业四大板块为主体的长白山绿色食品产业链。依托渤海商品交易所等电商平台，打造特色山珍电子营销新模式，打响长白山原生态、无公害高端品牌。七是新兴服务业。围绕落实全省服务业发展三年行动计划，大力实施信息与科技服务业振兴、节能环保服务业培育等“十大工程”，以重点行业率先突破引领全市服务业提质增效和转型升级，全年实现服务业增加值247亿元，增长8%。在物流、商贸、养老等重点领域选择一批示范企业先行先试，设立市本级服务标准化试点。加快双益农产品冷链物流园、星泰健康产业园等省级服务集聚区建设，推进14户重点企业非主营业务剥离，加快生产性服务业集聚发展。

　　（二）着力在项目建设上下功夫，增强绿色转型动力。坚持以增量调存量、扩总量、提质量，全力以赴扩大投资规模、优化项目结构、促进转型升级。

　　一是集中精力抓推进。全年计划安排重点项目450个，完成投资500亿元，其中亿元以上265个，完成投资440亿元；30亿元以上19个，完成投资160亿元。加快推进鼎运煤化工120万吨焦化产能等量替代、东北亚集团300万吨铁精粉等288个续建项目，开工中投微电子级高纯硅、德润玄武岩新材料等162个新项目，力争山水水泥、舜茂高纯二氧化硅二期等100个项目年内竣工。二是务求实效抓招商。创新招商模式，加快实现由依靠优惠政策招商向产业招商、由全员全方位招商向定点定向专业招商、由政府主导招商向政企联动招商“三个转变”。积极参加东北亚博览会，精心组织参与全国工商联6月份在我省举行的“民企吉林行”活动，超前谋划，主动对接，紧盯重点企业和地区，瞄准战略性投资和高附加值项目，大中选强、好中择优，引进区域性总部和功能性机构，主动承接发达地区产业转移。更加注重招商实效，切实提高项目合同履约率、资金到位率和落地开工率。全年引进市外资金增长10%，实际利用外资增长8%。三是积极主动抓争取。发挥绿色转型比较优势，抢抓政策机遇，把握投资导向，做好项目谋划包装和前期工作，建立健全项目推进机制，争取一批带动力强的基础设施、产业发展、社会事业、生态建设项目挤进国家和省盘子，千方百计争取更大份额，最大限度承接政策红利。全年新谋划3千万元以上项目260个，滚动谋划亿元以上项目100个以上。

　　（三）着力在生态宜居上下功夫，夯实绿色转型基础。坚持工业化与城镇化良性互动，生态建设与城镇化有机融合，积极稳妥推进以人为核心的生态城镇化。

一是完善城镇体系。科学规划顶层设计，群落式布局、节点式推进、特色化发展，实现中心城区、县城和重点城镇合理分工、功能互补、协同发展。积极推进产城融合，重点建设仙人桥、六道沟等一批产业强镇，打造百亿级经济重镇。加快实施我市主体功能区规划，支持示范城镇开展城乡建设用地增减挂钩试点，整合规划用地资源，提高用地效率。积极推进改善农村人居环境试点工作，有序推进新型农村社区建设。二是强化基础建设。新改建长白山大街西段等8条街路，完成金河大桥主体工程。推进供水、供热、物业改造和气化白山工程，市区新增燃气用户3千户，燃气普及率提高到41%。稳步实施城区老工业区和独立工矿区搬迁改造，加快改造临江大湖煤矿独立工矿区。营松高速竣工通车，鹤大高速完成主体工程，辉白高速完成征地及部分路基、桥涵、隧道工程，力争白临高速开工，加快建设大栗子至错草、白山至三道沟等干线公路，改建农村公路157公里，抓好错草至三道沟、靖宇至江源、临江至长白山机场三条干线公路以及市综合交通运输枢纽前期工作。积极推进四平、敦化至松江河，松江河至漫江和漫江至长白铁路项目。推进55个水利基础设施项目，完成矿泉湖水利枢纽主体工程。三是优化城乡面貌。大力开展环境卫生、城市牛皮癣、牌匾广告、占道经营等专项整治行动，强化园林绿化管理，实施翠柏广场、铁南街绿化改造工程。加快推进生活垃圾综合处理、垃圾焚烧发电以及矿欣路、长白山大街排水等工程，提升城市净化能力。实施库仓沟、碱厂沟河和浑江干流综合治理。推进市区城市管理综合执法试点工作，启动数字化城市管理平台建设，积极探索“两级政府、三级管理、四级网格”体系，加快构建市区一体化和精细化管理格局。高标准抓好100个村环境综合整治，解决6.5万农村人口安全饮水问题，建设“清洁美丽乡村”。四是推进生态城镇化。总结推广生态移民工作经验，按照“搬得出、留得住、能就业、有保障”要求，对林业生态保护区、矿泉水水源保护区、重点旅游景区、远离人口集聚区的贫困村屯，实施整村搬迁和地质灾害搬迁；对林区散居户、无耕地农民等重点移民对象，通过政策引导自愿搬迁；对资源枯竭型工矿区和国有林场所在地，通过合并和搬迁，向中心城镇或相邻城镇就地就近整体迁移。分类有序引导农民进城安居置业，争创国家新型城镇化试点市。五是筑牢生态屏障。大力实施长白山林区生态系统修复工程，清收林地14.5万亩、封山3万亩，人工造林和森林抚育各2.5万亩，创建老虎山国家级森林公园。加强重点流域治理和大气污染防治，将PM2.5纳入空气质量监测体系，全年削减化学需氧量950吨、氨氮248吨，“三江”干流出境水质保持国家3类水体标准。积极推进煤矸石、粉煤灰、低品位硅藻土、木材剩余物等资源化综合利用项目。完成土地复垦和矿山地质环境恢复省定任务。

　　（四）着力在改革开放上下功夫，激发绿色转型活力。勇于破除制约瓶颈，加快释放改革开放红利，为各类市场主体创造更为平等、公平竞争的市场环境。

　　一是深化各项改革。深入推进重点领域和关键环节改革，确保12方面经济体制和17方面生态文明体制142项改革任务按期完成。继续推进政府职能转变和机构改革，实行“四个一律”。强化预算管理改革，建立现代公共财政制度。积极稳妥推进农村土地流转改革，全市40%乡镇开展土地确权登记颁证。推进集体林权制度配套改革，做好国有林场改革前期准备工作。二是扩大对外开放。积极推进长白国家重点开发开放试验区和边境经济合作区建设，筹建临江边境经济合作区，力争早日获批。抓好长白、临江过境通道前期工作，探索建设内陆港和保税物流园区，设立外贸扶持资金，争取出口退税政策。积极发展边境地区民间贸易。发挥国家级外贸转型示范基地和省级林木深加工产品出口基地带动作用，扶持恒大冰泉、舜茂科技、金隆木业等龙头企业扩大出口规模，优化外贸结构，培育新增长点。主动融入长吉图开发开放先导区和东北东部区域合作，扎实推进基础设施、产业布局、政策规划、要素市场一体化。三是壮大民营经济。大力实施百户小微企业帮扶和百户重点民企转型 “双百工程”，围绕“五大工程”，出台支持力度更大的政策措施，确保“五项工作机制”和“三项扶持保障制度”有效运转。完善市创业大厦和孵化基地服务功能，推动各县（市、区）加快建设中小企业服务平台，实现省、市、县互联互通、信息共享。培育本土企业发展壮大，支持本市资本二次投资，鼓励本地人才二次创业，迅速掀起大众创业、草根创业新浪潮。全年民营经济主营业务收入达到1600亿元，从业人员达到37万人，民企和个体工商户达到8千和7.3万户，分别增长14.3%、5%、8%和7.4%。

　　（五）着力在财税金融上下功夫，强化绿色转型支撑。进一步规范财税管理，推进金融创新，发挥杠杆作用，为绿色转型发展提供强力支持。

　　一是推进增收节支。培植税源企业，争取政策支持和转移支付，切实提高财政收入可持续增长能力。强化税源监控，探索构建“以地控税、信息比对、重点实测、动态监控”征管新机制，确保应收尽收。调整优化财政支出结构，压缩一般性支出，将有限财力更多用在刀刃上。进一步加大预算决算、“三公”经费和审计信息公开力度，加强财政资金监管和政府性债务管理，严格防范债务风险。二是拓展融资渠道。深化政银企合作，协调域内银行不断创新担保抵押方式和金融产品，加大信贷投放力度。探索组建股权、林权、土地经营收益权和矿权等产权交易平台，提高资源市场化程度。积极引导天使投资、风险投资、股权基金等流向绿色经济，提高资产证券化率，降低企业融资成本，优化资源配置，力争万良人参市场、喜丰节水科技年内上市。三是完善金融体系。积极引进商业银行，支持金融机构加强服务网点建设。深化农村信用联社改革，加快推进临江农商行和江源、临江、长白等地村镇银行建设。整合担保资源，提升担保能力，鼓励设立金融中介机构。促进证券、保险行业健康发展，启动森林火灾保险试点，增加特色农业险种，进一步扩大政策性农业保险覆盖范围。强力推进信用白山建设，完善资本市场诚信监管制度，严厉打击非法集资、恶意逃废债，改善金融生态环境。

　　（六）着力在和谐稳定上下功夫，强化绿色转型保障。继续把改善和保障民生作为工作的出发点和落脚点，进一步加大公共财政投入力度，切实办好20件民生实事。

　　一是均衡发展社会事业。大力实施学前教育推进工程, 优化普通高中资源配置，提升特殊教育和民族教育办学水平，完成义务教育均衡发展阶段任务。加快建设现代职业教育体系，推进长白山职业技术学院和旅游职教集团建设，为转型发展提供职业人才支撑。强化医疗卫生体系建设，推进第二批县级公立医院综合改革，巩固完善基本药物制度和基层公共卫生新机制，鼓励社会办医，全力做好重点传染病防控工作。加强人口计生工作，实施优生服务工程，提高出生人口素质。全面推进文化惠民工程，抓好“两节”、免费开放、文化下乡等公益性文化活动。广泛开展全民健身活动，深化教体结合，积极备战第十三届全国冬运会。投资6.1亿元加强“智慧白山” 和铁塔公司 4G网络建设，实施智慧强政、兴业、惠民项目48个。二是提升社会保障能力。大力实施就业创业工程，开展全民创业促就业等系列活动，城镇新增就业4.2万人，农村劳动力转移就业11万人。加大五大险种扩面征缴力度，加快构建统一的城乡居民基本养老保险制度，全市社保“一卡通”覆盖率达到53%，医保信息服务平台实现全覆盖。完善社会救助体系，进一步提高城乡低保、大病救助、社会福利和救灾救济水平，完成全国普惠型儿童福利制度建设试点任务，新殡仪馆和残疾儿童康复中心投入使用。积极推进保障性安居工程，改造城市和国有工矿棚户区28.7万平方米。三是维护社会和谐稳定。继续加大信访积案化解力度，健全涉法涉诉信访导入司法机制，信访案件和人数分别下降10%、20%。以法治为引领，深入推进平安建设，完善以“六张网”为骨架的打防管控一体化治安防控体系，力争可控性案件下降5%以上，进一步提升人民群众的安全感和满意度。大力实施强基富民固边、兴边富民和民族团结创建活动，再创全国双拥模范城，维护边境地区和谐稳定。深入开展“六五普法”、社区矫正规范化建设年和法律援助阳光工程，推进行政大复议试点工作，切实提高全民法律素质和依法治理水平。四是筑牢安全生产防线。全面宣传贯彻新《安全生产法》，深入推进安全监管防控体系建设，高危企业标准化建设百分之百达标，网格化管理实现全覆盖。加快煤炭行业整合重组步伐，推进小煤矿关闭退出。建设市食品检验检测中心和食品药品安全投诉举报中心。持续开展大检查大整改，加大重点行业领域隐患排查治理力度，严厉打非治违，严格责任追究，坚决遏制重特大事故发生。

　　（七）着力在法治政府建设上下功夫，凝聚绿色转型合力。坚持法无授权不可为，法定职责必须为，加快建设职能科学、权责法定、执法严明、公开公正、廉洁高效、守法诚信的法治政府。

　　一是全面推进依法行政。深入贯彻依法治国重大决定及依法治省、治市实施意见，着力提高运用法治思维和法治方式深化改革、推动发展、维护稳定的能力，建设法治政府。依法履行政府职能，推进机构、职能、权限、责任法定化。健全依法决策机制，建立重大决策终身责任追究、责任倒查等纠错问责制度，完善公众参与政府决策机制和政府法律顾问制度。深化行政执法体制改革，细化、量化行政裁量标准，向改革要效益，靠管理求效率。二是强化权力监督制约。完善监督体制机制，确保所有行政权力有法可依、公开运行。自觉接受市人大、政协监督，认真办理建议提案，及时将合理意见纳入政府决策。主动接受法律监督、舆论监督和社会监督，让权力在阳光下规范运行。深入实施“权力入笼”工程，全面推进政务公开，落实权力清单制度，实行“一表制”和“并联审批”，精简审批事项，重构审批流程。健全对关键部门和重点岗位的监督制约机制，防止权力滥用。完善审计制度，对公共资金、国有资产、国有资源和领导干部履行经济责任情况实行审计全覆盖。三是持续加强作风建设。强化制度建设，深化“四风”整治，巩固和拓展群众路线教育实践活动成果，践行“三严三实”，以清廉之行，谋民生之福。厉行勤俭节约，反对铺张浪费，严控“三公”经费支出，全面推进公车改革。持续改进工作作风，规范公正文明执法，进一步优化经济发展软环境。完善惩防体系，坚决查处违纪违法案件，加强源头治腐，形成“不敢腐、不能腐、不想腐”的有效机制，营造廉洁奉公、风清气正的良好政风。四是切实提高服务能力。讲党性、讲原则、讲诚信，重细节、重程序、重成效。加强学习型政府和研究型队伍建设，问需于民、问计于民、问政于民，做到干部工作在一线、政策落实在一线、问题解决在一线、成效检验在一线。严格执行招商引资、项目建设、民生实事等“作业单”制度，加强量化考核和过程控制，实施“会诊式”督查，推动任务、责任、进度、绩效“四落实”，努力形成敢于定事、勤于干事、善于成事的强大合力。

　　各位代表！改革创新风正劲，绿色转型正当时。让我们在省委、省政府和市委的坚强领导下，振奋精神、凝心聚力、锐意进取，在新的征程上奋力谱写白山绿色转型发展新篇章！
